# Human Monkeypox: Fifty-Two Years based analysis and Updates

**DOI:** 10.12669/pjms.38.6.6775

**Published:** 2022

**Authors:** Sultan Ayoub Meo, Shaukat Ali Jawaid

**Affiliations:** 1 Sultan Ayoub Meo, Department of Physiology, College of Medicine, King Saud University, Riyadh, Saudi Arabia; 2 Shaukat Ali Jawaid, Chief Editor, Pakistan Journal of Medical Sciences, Karachi, Pakistan

**Keywords:** Human Monkeypox, Outbreak, Prevalence

## Abstract

The growing prevalence of human monkeypox infection has developed an alarming situation worldwide. Monkeypox virus was first time found in 1958 in monkeys and later spread to humans. The first case of human monkeypox was reported in September 1970 in the Democratic Republic of the Congo. Human monkeypox was found outside Africa in the year 2003 in United States. More recently, from May 7 2022 to June 29, 2022, the monkeypox cases are swiftly spread worldwide, involving over 50 countries, and affecting 5115 people in Europe, the United Kingdom, North America, and South America, Asia, Australia, and the Middle East. The confirmed monkeypox cases in the United Kingdom from May 7, 2022 to June 29, 2022, are 1076 (21.03%); Germany 874 (17.08%); Spain 800 (15.64%); France 440 (8.60%); Portugal 391 (7.64%); United States 350 (6.84); Canada 276 (5.39%); Netherlands 257 (5.02%); Italy 159 (3.10); Belgium 117 (2.28%); Switzerland 81 (1.58%); Israel 33 (0.64%), and Ireland 31 (0.60%). However, in about 35 countries, the cases are less than 20 in each country. The epidemiological trends of the human monkeypox infection are swiftly shifting from endemic regions to non-endemic countries. The global health authorities must take priority-based preventive measures to stop the outbreaks of monkeypox disease across the globe.

## INTRODUCTION

Monkeypox is a zoonotic disease, caused by a virus known as the monkeypox virus (MPXV). MPXV is a “double-stranded DNA virus” belonging to the genus Orthopoxvirus, subfamily Chordopoxvirinae and family poxviridae.^1^-^4^ Monkeypox was first found in 1958 after the occurrences of a pox-like disease in monkeys, hence the disease became popular with the name monkeypox. The first case of human monkeypox was reported in 1970 in the Democratic Republic of the Congo. After that, the disease has been found in other central and western African countries.^1^ More recently, since May 7, 2022, human monkeypox cases are reported in European countries, the United Kingdom, the United States, Australia, the Middle East and many other states worldwide.^5^,^6^

### Biological characteristics

Monkeypox (MPX) virus has distinctive surface tubules and a dumbbell-shaped core component. The monkeypox virus is brick-shaped, with a size of approximately 200-250 nm. The virus is enclosed by a “lipoprotein envelope with a linear double-stranded DNA genome”.^2^,^4^ There are two distinct genetic clades of the monkeypox virus; the Central African clade “(Congo Basin clade)”, and “The West African Clade”. The Central African clade is a more transmissible and severe disease impact.^5^ The Central African clade is more deadly, with a case fatality rate of about 11% in the unvaccinated population, however, the West African clade has a better prognosis with less than 1% fatality rate. The Central African Clade originated from Gabon, Cameroon, Republic of Congo (ROC), Central African Republic (CAR), Sudan, and the Democratic Republic of Congo (DRC). West African clade originated from outbreaks in Nigeria, Liberia, Ivory Coast, Sierra Leone, and the USA (imported from Ghana) 1,5.

### Transmission of monkeypox virus

MPXV can transmit from animal to animal, animal to human, and person to person. The virus can spread through direct contact with body fluids, cutaneous, mucosal lesions or sores on an infected person or with materials that have touched body fluids or sores, such as clothing or linens. It can also be spread by respiratory, oral, and nasal secretions, and during skin-to-skin, handshake, hugging, kissing, and sexual contact. The virus can cross the placenta from the mother to the fetus.^7^,^8^

Antinori et al., 2022^9^, reported that the transmission of the infection is mainly through close contracts at home, office, community gatherings, healthcare workers, or sexual contacts. The authors further reported that the disease transmits on community contacts: household (23%), sexual (22%), friend/shared space (25%), workplace (23%), and community healthcare (7%). The literature suggests that transmission occurs through sexual contact. The authors further found four cases of young adult men in Italy, the seminal fluid samples were positive for monkeypox viral DNA. These reports further demonstrate that there is a high possibility of sexual transmission of the monkeypox virus.

### Global Epidemiology of monkeypox disease

The first case of monkeypox was found in 1958 in monkeys after the occurrences of a pox-like disease in monkeys, hence the disease became popular with the name monkeypox. In the Democratic Republic of the Congo, the first human monkeypox case was reported in 1970. After that, the cases have been found in other central and western African countries.^1^ The literature highlights that “African rodents, squirrels, mice, rats, dogs, and monkeys are the natural reservoir and cause infections in humans”.^10^ There are two genetically clades, Congo Basin (Central African) clade more frequently occurred than the West African clade and caused human-to-human transmission.^11^,^12^

Human monkeypox cases have been reported outside of Africa in early 2000. In 2003, Gambian rats imported from Ghana infected 53 people with monkeypox in the Midwestern United States.^12^ In October 2018, a man was infected who travelled from Nigeria to Israel.^13^ In May 2019, another case travelled from Nigeria to Singapore.^14^ More recently, 2022. the world is witnessing the monkeypox virus causing a multi-country outbreak on almost all the continents outside Africa. 10,12

Since May 7, 2022, monkeypox cases have been reported in many European countries, the United Kingdom, the United States, Australia, the Middle East and other states in the world.^5^,^6^ The worldwide total number of confirmed and suspected cases of human monkeypox infection from 1970 to June 29, 2022, is 46,247 (Table-I). The number of confirmed cases of human monkeypox is 2805 and suspected cases are 38327. Among the confirmed cases 2,498 are of the Central African Clade, 307 are of the West African Clade (Table-I), and 5115 are in other 50 countries in Europe, the USA, the UK, and the Middle East and Australia (Table-II).^4^-^6^, ^11^-^12^

**Table I:** Monkeypox confirmed and suspected cases from 1970 to June 29, 2022.^4^-^6^,^10^-^12^

The time in decades/years	Central African Clade	West African Clade	Total
***A: Confirmed cases***			
1970-1979	38	9	47
1980-1989	355	1	356
1990-1999	520	0	520
2000-2009	92	47	139
2010-2019	85	195	280
2021	--	34	34
2022 (Jan-June)	1408	21	1429
Confirmed Cases	2498	307	2805
B: Suspected Cases			
2000-2009	10,027	--	10,027
2009-2019	18,788	--	18788
2020	6,257	--	3189
2021	3,091	98	66
2022 (Jan-June)	--	66	66
Suspected Cases	38,163	164	38,327
Total Confirmed & Suspected Cases	40,661	471	41,132
C: Recent cases in Europe, UK, USA, Australia, Middle East			5,115

Grand Total cases			46,247

**Table II:** Monkeypox confirmed cases in Europe, UK, USA, Australia and the Middle East from May 7 to June 29, 2022.^6^ (n= number of countries).

Country	Cases June 29 2022 (n=50)
United Kingdom	1076 (21.03%)
Germany	874 (17.08%)
Spain	800 (15.64%)
France	440 (8.60%)
Portugal	391 (7.64%)
United States	350 (6.84%)
Canada	276 (5.39%)
Netherlands	257 (5.02%)
Italy	159 (3.10%)
Belgium	117 (2.28%)
Switzerland	81 (1.58%)
Israel	33 (0.64%)
Ireland	31 (0.60%)
Brazil	21 (0.41%)
Austria	20 (0.39%)
Denmark	18 (0.35%)
Norway	15 (0.29%)
Mexico	15 (0.29%)
Sweden	13 (0.25%)
United Arab Emirates	13 (0.25%)
Hungary	12 (0.23%)
Australia	10 (0.19%)
Poland	10 (0.19%)
Slovenia	9 (0.17%)
Romania	9 (0.17%)
Czechia	8 (0.15%)
Slovenia	7 (0.13%)
Chile	6 (0.11%)
Finland	4 (0.07%)
Argentina	4 (0.07%)
Romania	4 (0.07%)
Norway	4 (0.07%)
Iceland	4 (0.07%)
Greece	3 (0.05%)
Benin	3 (0.05%)
Colombia	3 (0.05%)
Peru	3 (0.05%)
Hungary	3 (0.05%)
Bulgaria	2 (0.03%)
Lativa	2 (0.03%)
Morocco	1 (0.01%)
Malta	1 (0.01%)
Gibraltar	1 (0.01%)
Venenzuela	1 (0.01%)
Luxembourg	1 (0.01%)
Bahamas	1 (0.01%)
Croatia	1 (0.01%)
Estonia	1 (0.01%)
Georgia	1 (0.01%)
Serbia	1 (0.01%)
Singapore	1 (0.01%)

Total	5115

Presently, monkeypox cases are swiftly spread and involved many countries worldwide. These cases are 5,115 which started in the United Kingdom on May 7, 2022, and spread over 50 countries in Europe, North America, South America, Asia, Australia, and the Middle East. The confirmed monkeypox cases in the United Kingdom from May 7, 2022 to June 29, 2022, are 1076 (21.03%); Germany 874 (17.08%); Spain 800 (15.64%); France 440 (8.60%); Portugal 391 (7.62%); United States 350 (6.84); Canada 276 (5.39%); Netherlands 257 (5.02%); Italy 159 (3.10); Belgium 117 (1.59%); Switzerland 81 (1.58%); Israel 33 (0.64%), Ireland, and 31 (0.64%) (Table II). However, in other countries, the cases reported are less than 25. The epidemiological trends of the human monkeypox virus are swiftly shifting from endemic regions to non-endemic countries.

### Clinical Characteristics

Once the virus enters the body, replicates at the site and spreads to local lymph nodes. After that, viremia spread to body organs. This signifies the incubation period of 7-14 days with a maximum period of 21 days. The clinical features start with a secondary viremia leading to one to two days of prodromal clinical features such as fever and lymphadenopathy. The skin lesions start in the oropharynx and then appear in other parts of the body.^15^, ^16^ The “lesions begin as macules or papules, which progress to pustules and umbilicated vesicles and ulcers and eventually to crusted scabs”.^17^ The condition may establish differential diagnoses of the diseases including “syphilis, chancroid, varicella zoster, herpes simplex, hand-foot-and-mouth disease, and molluscum contagious”. The patients are infectious from the onset of symptoms until all scabs have been resolved.^18^

The common presenting symptoms include “fever, oral sores, swollen lymph nodes, and pain when swallowing”. The skin rashes “vesicular, pustular or ulcerated lesions sometimes appear first without spreading to other parts of the body” In some patients, pustules appear before the symptoms like fever.^5^ The clinical characteristics of “monkeypox” infection mostly consisted of mild symptoms, patients complained of headache, body ache, generalized myalgia, malaise and acute onset of fever. The skin lesions start as “macules or papules, which progress to pustules, umbilicated vesicles, ulcers and eventually to crusted scabs.^11^”

Diagnosis and Clinical Management

“Taking care of the patients with suspected or confirmed monkeypox requires early recognition through screening protocols adapted to local settings, prompt, isolation and rapid implementation of appropriate infection prevention and control (IPC). Measures.” As regards therapeutics, Tecorivimat is an antiviral drug which has recently been approved by the European Medicines Agency for orthopovirus-associated infections, including monkeypox. It is based on animal models and data for safety, pharmacokinetics and pharmacodynamics in humans. It is hoped that reliable and interpretable results on its safety and efficacy will be available soon.^19^

## CONCLUSION

The epidemiological trends of the human monkeypox virus are swiftly shifting from endemic regions to non-endemic countries. Worldwide, the total number of confirmed and suspected cases of human monkeypox from 1970 to June 29, 2022, is 46,247. More recently, from May 7 2022 to June 29, 2022, the monkeypox cases are swiftly spread worldwide, involving over 50 countries, and affecting 5115 people in Europe, the United Kingdom, North America, and South America, Asia, Australia, and the Middle East. The transmission of diseases is mainly animal to human, and human to human. The major clinical characteristics of “monkeypox” infection are headache, body ache, generalized myalgia, malaise, acute onset of fever, swollen lymph nodes, and skin lesions. The skin lesion starts as macules or papules, progresses to pustules, vesicles and ulcers, and is ultimately converted into crusted scabs. The global health authorities must take priority-based preventive measures to stop the outbreaks of monkeypox disease across the globe. The education of the public, patients, and physicians is of the utmost importance to fight against monkeypox and stop it to become a pandemic.
